# Implementation of recommended type 2 diabetes care for people with severe mental illness – a qualitative exploration with healthcare professionals

**DOI:** 10.1186/s12888-016-0942-2

**Published:** 2016-07-08

**Authors:** Hayley McBain, Kathleen Mulligan, Frederique Lamontagne-Godwin, Julia Jones, Mark Haddad, Chris Flood, David Thomas, Alan Simpson

**Affiliations:** School of Health Sciences, City University London, Northampton Square, London, EC1V 0HB UK; East London NHS Foundation Trust, 9 Alie Street, London, E1 8DE UK

**Keywords:** Qualitative, Theoretical domains framework, Type 2 diabetes, Severe mental illness, Barriers, Facilitators, Comorbidity, Health behaviours

## Abstract

**Background:**

The purpose of this study was to explore the barriers and facilitators healthcare professionals experience when managing type 2 diabetes in people with severe mental illness (SMI).

**Methods:**

A qualitative semi-structured interview approach was employed. Questions were structured according to the Theoretical Domains Framework (TDF), which outlines 14 domains that can act as barriers and facilitators to changing behaviour. Interviews were audio-recorded and transcribed verbatim. The data were coded according to the 14 domains of the TDF, belief statements were created within each domain and the most relevant belief statements within each domain identified through a consensus approach. Analyses were conducted by two researchers, and discrepancies agreed with a third researcher.

**Results:**

Sixteen healthcare professionals, from a range of services, involved in the care of people with type 2 diabetes and SMI took part in an interview. Inter-rater reliability for each of the domains varied (25 %-74 %). All fourteen domains were deemed relevant, with 42 specific beliefs identified as important to the target behaviour. Participants identified having relevant knowledge and skills for diabetes management, prioritising this area of health, and reviewing health behaviours to develop action plans, as particularly important. At an organisational level, integrated care provision and shared information technology (IT) services between mental health and physical services, and clearly defined roles and responsibilities for the different professions, with designated time to undertake the work were identified as crucial.

**Conclusions:**

The findings highlight that healthcare professionals’ experience a range of barriers and enablers when attempting to manage type 2 diabetes in people with SMI. These include organisational factors and individual beliefs, suggesting that interventions need to be targeted at both an organisation and individual level in order to change behaviour. Further work is needed to model these relationships in a larger sample of participants in line with the MRC guidance for developing complex interventions.

**Electronic supplementary material:**

The online version of this article (doi:10.1186/s12888-016-0942-2) contains supplementary material, which is available to authorized users.

## Background

The prevalence of diabetes is increasing globally, affecting an estimated 8.8 % of adults – 415 million people worldwide – and accounting for 12 % of international health expenditure [1,1]. In the UK, 6.2 % of the adult population are estimated to have diabetes and as in other high income countries around 90 % of have type 2 diabetes [1]. Although there is no single cause of the condition, one important risk factor is diagnosis of a severe mental illness (SMI), such as schizophrenia, bipolar disorder and other psychoses. Research indicates a 2–3 fold increased incidence of type 2 diabetes among people with SMI [2]. This increased risk has been attributed to a number of factors, including the effects of anti-psychotic medications [3], lifestyle factors such as poor diet, obesity and physical inactivity [2, 4] and high rates of smoking [5]. As a consequence, in people diagnosed with diabetes, those with SMI have been found to die significantly younger than people without SMI [6] and experience a greater risk of diabetes complications that require specialist treatment [7].

These significant health inequalities could be explained by the inequity of diabetes care between people with and without SMI. People with SMI are reported to be less likely to receive recommended diabetes care, including retinopathy screening; foot examinations; HbA1c, renal and cholesterol checks; and diabetes education. They are also less likely to meet recommended blood glucose targets than those without SMI [8–13]. Although some studies have failed to find any significant differences in diabetes care between people with and without SMI [14–18], this has been attributed to the greater number of medical visits made by people with SMI compared to those without SMI [14, 15], a bias towards data that includes only service users who are registered with a general practitioner (GP) [18] or studies that recruit primarily male veterans [13–16] making generalisability of these latter findings problematic.

The reasons for these disparities in care are largely unknown, which means that the development of interventions to ensure that people receive recommended care are not yet possible. Care pathways for people with a SMI and diabetes are often complex and fragmented, yet co-location of mental and physical services seems to have had variable impact - increasing the likelihood of receiving diabetes-related foot examinations for example, but having no effect on rates of retinopathy screening [19]. There also appears to be confusion and role ambiguity about responsibility for monitoring and supporting type 2 diabetes in SMI, between primary care and specialist mental health care services [20]. This is despite the fact that guidelines for the management of psychosis and schizophrenia state that specialist mental health teams should assume the role of monitoring service users’ physical health for at least the first 12 months or until the person’s condition has stabilised, thereafter responsibility should be transferred to primary care [21]. The knowledge and skills of mental health nurses to deliver and support diabetes care has also been questioned [20, 22, 23].

The purpose of this study was to explore, using an established theoretical framework, a broad range of barriers and enablers that may affect the practice of primary care, specialist mental health and diabetes specialist clinicians in their management of type 2 diabetes in people with SMI. It is hoped that the study will increase knowledge and understanding of individual and organisational level interventions that may support healthcare professionals in this endeavour and inform further research on this important topic.

## Methods

### Design and sampling

A qualitative study, involving semi-structured one-to-one interviews with healthcare professionals involved in the treatment and care of people with type 2 diabetes and SMI, working within an inner London service provider, were eligible to participate. A combination of snowball and purposive sampling techniques were used to recruit participants. Interviews ceased when saturation of themes was achieved, defined as the emergence of no new themes in relation to the research question. In practice, this meant that the interviewer had a sense that nothing new was emerging from the interviews. Coding then began to explore the reality of this [24].

### Data collection

An interview topic guide was developed based on the Theoretical Domains Framework (TDF) for behaviour change [25]. This framework proposes 14 theoretically distinct domains, each of which is composed of psychological constructs that have been found to influence behaviour across a range of different contexts. The interview schedule consisted of 24 questions (see Additional file 1). Prompts were included to address the specific constructs within each domain.

We initially identified 2 key healthcare professionals involved in caring for people with type 2 diabetes and SMI; these participants were then asked to identify two individuals who they believe would provide useful information, and so on until the full sample were recruited. Of those identified in this way eligible healthcare professionals were emailed the information sheet and consent form prior to the interview.

Interviews were conducted between October 2014 and June 2015. Interviews took place at the participants’ place of work and lasted between 15 and 50 min (mean = 31 min, SD = 8.4 min). All interviews were digitally recorded, with the participants’ permission, and transcribed verbatim by a professional transcription company. Any identifiable data within the transcripts were anonymised.

### Data Analysis

The analysis of the transcripts was performed using the TDF, in the following five steps [26, 27].

1. Coding interview transcripts: Two researchers (HM and FLG) independently coded all interview transcripts into the 14 domains, manually. The two coders then came together to compare results. Any differences between the two coders were then discussed until consensus was reached. If consensus could not be reached a third researcher (KM) was consulted. This process was undertaken whilst the interviews were being conducted and informed the decision to cease recruitment due to data saturation. Using established methodology [27] reliability was determined across the final four interviews (prior to consensus being reached) by calculating the percentage agreement/disagreement, to measure consistency in coding within and across the domains [28, 29]. Complete agreement was defined as the two coders identifying the same response and coding it into the same domain. Partial agreement was defined as the two coders identifying the same response, but coding it into different domains. Complete disagreement was defined as only one coder identifying a response and coding it into a domain, but not the other coder.
2. Generating specific beliefs: A final coded version of each transcript was uploaded into QSR NVivo 10. Specific belief statements were then generated by one of the researchers (HM), these statements represented the underlying belief represented in each response. A belief statement was created for each response, moving through all responses within a domain. Responses with similar underlying themes were coded under the same belief statement. Otherwise a new belief statement was created. Responses that were similar in their underlying theme, but were polar opposites e.g. “I know how to manage diabetes in someone with SMI” and “I do not know how to manage diabetes in someone with SMI” were grouped together. The belief statement was worded to convey a meaning that was common to multiple utterances, therefore the wording of the belief statements was an iterative process.
3. Frequency of beliefs and responses: In order to identify the most frequently reported barriers and enablers of the targeted behaviour a frequency count for each belief statement, representing the number of participants who mentioned the belief, was calculated together with the frequency of responses per domain.
4. Relevant belief statements within each domain were identified through consensus discussion within the research team. These decisions took into account four factors concurrently (i) frequency of the belief across interviews; (ii) presence of conflicting beliefs; (iii) perceived strength of the beliefs impacting upon management of type 2 diabetes in people with SMI and (iv) identification of beliefs that had been reported elsewhere in the literature.
5. Mapping constructs onto specific beliefs: The belief statements that were deemed relevant within each of the 14 domains were then coded back into the TDF domains to ensure accuracy. Two independent coders, with experience of using the TDF and blinded to the domain in which the belief statement had been created, were asked to assign a TDF domain to each belief statement. Level of agreement was summarised as the number of coders who agreed divided by the total number of coders.

Whilst the use of numbers to quantify qualitative results can be contentious [30], in presenting our results below we have chosen to include some indication of the numbers of participants making belief statements in relation to particular domains. This is in line with previous use of the TDF [26, 27, 31].

We have incorporated numbers in our qualitative research to document and verify the researcher interpretations of the data [32] and specifically in line with two approaches identified by Maxwell [30]. First, the use of numbers contributes towards ‘internal generalizability’ or generalization within the collection of individuals studied, establishing that the themes or findings identified are characteristic of this set of individuals as a whole. Secondly, numbers enable us to identify and correctly characterize the diversity of actions, perceptions, or beliefs in the setting or group studied, reducing the risk of bias towards seeking uniformity and overlooking diversity.

## Results

### Participant characteristics

Twenty healthcare professionals were approached to take part and a total of 16 (80 %) interviews were undertaken, at which point saturation of themes was established. The sample included psychiatrists (*n* = 4), community mental health nurses (*n* = 4), diabetes specialist nurses (*n* = 2), a diabetologist (*n* = 1), GPs (*n* = 3), a practice nurse (*n* = 1) and a primary care liaison nurse (*n* = 1). A majority of the participants were female (*n* = 10, 62.5 %) and on average had 19 years (SD = 7.9) in clinical practice.

### Inter-rater reliability

For the last four interviews, the interrater agreement between the two coders across the 14 domains ranged from 25 % to 74 %. When blinded researchers were asked to map the belief statements onto a domain, for 86 (65.6 %) of the 131 beliefs both researchers mapped the belief statement onto the intended domain, for 35 (26.7 %) beliefs only one researcher mapped the belief statement onto the intended domain and for 10 (0.08 %) belief statements there was zero agreement.

### Domains

All domains were mentioned by at least seven participants. *Knowledge*, *Social Professional Role and Identity*, *Beliefs about Consequences*, *Goals*, *Environmental Context and Resources* and *Social Influence* were mentioned by all 16 participants (Table 1). All domains were supported by at least 10 quotes, the domains with the most quotes were *Environmental Context and Resources*, *Social Professional Role and Identity* and *Social Influences. Intentions* were mentioned by the fewest number of participants and *Beliefs about Capabilities* supported by the least number of quotes.

**Table 1:** Belief statements and sample quotes for each domain

Domain	Specific belief	Sample quote	No. of participants	Total no. of quotes
Behavioural Regulation	I have a plan, either in my team or on my own, when managing diabetes in someone with SMI.	*"…within the CPA [Care Programme Approach] document there is a bit that is supposed to be filled in, I’d do a slightly different, what do you call it, aid memoir to go through [the Care Programme Approach document]. And on that I’ve got diabetes, exercise, diet, smoking, all of that, which I would see as part of that."*	5	8
	I review how I manage diabetes in people with SMI, and identify ways in which I can improve.	*"Diabetes is always on the agenda in teams and even the nurses meetings with, there was a presentation about one patient, one incident, someone had diabetes and how that should have been managed. So it’s always spoken about, our action plans, how should we manage it? How did we not manage it properly last time, and how should we improve? So that is always on the agenda for the nurses, yeah."*	3	3
Beliefs about Capabilities	I feel confident about managing diabetes in people with SMI.	*"[Managing diabetes in people with SMI is] …well, within the boundaries of what I can do."*	9	10
Beliefs about Consequences	If I didn't take steps to manage diabetes in someone with SMI, they would come to serious harm.	*“I think not until you’ve seen one of the worst cases of diabetes would you want to start taking it seriously. Because I think I have seen cases where people have had their legs amputated and it gets you back on your BM.”*	13	22
	Poorly controlled diabetes affects a person’s mental health.	*“One thing that’s very obvious from looking after mental health patients, there’s a definite correlation between high blood sugars and changes in their mental health state. There’s no question about it. It’s obvious. We’ve read about it. We’ve read all the research, when you’re looking after somebody. And it soon becomes very apparent to the ward staff. If they have someone whose sugars are high their mood is going to change. If their sugars are low their mood is going to change.”*	9	9
Emotion	Managing type 2 diabetes in people with SMI worries or concerns me.	*“For me I think [managing diabetes in someone with SMI] worries me in, it worries me, it does worry me, no, I’m concerned rather than worried to be honest.”*	10	13
	Managing type 2 diabetes in people with SMI frustrates me.	*“Well I suppose it can be frustrating for staff if people are, despite all the prompting and education, people just want to do their own thing, the patients say, well I don’t, I don’t really care actually, I don’t care what the long term consequence is, and I just want to have my cake today really, do you know what I mean, I suppose that can be a bit frustrating.”*	3	5
	Working with people with SMI scares me.	*“We’ve all got fear of mental health.”*	1	1
Environmental Context & Resources	I have access to a GP to help me manage diabetes in people with SMI.	*“…if anyone came here and had diabetes and there were issues I would just be picking the phone and calling the GP and saying, so and so is here, these are the problems, can they come and see you or book an appointment?”*	13	31
	More integrated IT systems would make it easier for me to manage diabetes in people with SMI.	*“Last week I think we spent nearly three of our days, our evenings catching up [the IT system] was down from a Thursday until Monday lunchtime so everything had to be backed up and then it’s got to be added on to [the IT system] and that has big consequences. Not only when that when you’re seeing that person because you’re limited on what information you can get. But it’s actually documenting for staff who have been on the ward over the weekend and can’t access. They haven’t got a care plan so they’ll ring me and say do you have the email of the care plan because we can’t access [the IT system].”*	10	18
	I don't have enough time to manage diabetes in people with SMI.	*“I think sometimes probably the resources, sometimes it can be very busy and very stretched and you may not have the time to dedicate and evaluate someone who’s very complex.”*	10	15
	I have access to someone with specialist mental health knowledge to help me manage people with diabetes and SMI.	*“I think more integrated working would encourage me, so at the moment we have a local enhanced service, it’s just local to [the area] where we have a CPN who comes in on a once every five weeks basis and does clinics here with her mental health patients and although I don’t see them with her jointly, the fact that she is in the building, we talk about other cases and that’s really handy.”*	6	7
	I have access to people with specialist diabetes knowledge to help me manage diabetes in people with SMI	*“…we have an excellent diabetic nurse actually, who’s so hard working and very proactive, and she will often email me if she’s concerned about a patient with a plan, she communicates very well, she emails the nurses, put it’s on [the IT system] and will make sure that I know and the ward doctor knows as well, so that the communication is very robust.”*	11	27
Goals	Managing patients' type 2 diabetes is as important as managing their mental health.	*“There is really no room for negotiation with that because if you’re looking after a patient you’re not only looking after the head or the brain but you’re looking after everything about them, about that particular patient. And with diabetes being one of them it’s just like vulnerability, you can’t take one and leave the other.”*	5	7
	Diabetes goals and targets need to be tailored for people with SMI.	*“You’ve got to set realistic goals not try and set goals that are not achievable because that’s setting people up to fail. So you have to tailor your goals and targets to that person sitting in front of you.”*	5	6
	There is a definite focus in my trust on managing type 2 diabetes in people with SMI.	*“…if you look at that teaching session that’s going on today down at [the hospital], physical health is much more on the agenda I think.”*	4	5
	I prioritise management of mental health over physical health in people with type 2 diabetes and SMI.	*“I think the risk is that very often the diabetes medication, because it isn’t an acute illness that has to be treated now, it’s something that sometimes gets left and just put on the back burner a bit while we maybe, let’s get the more psychologically stable first and then we can address the diabetes which very often might delay good treatment by years.”*	3	7
Intentions	I intend to follow NICE diabetes guidelines for patients who have type 2 diabetes and SMI	*“I’m going to refer her to get her DRI, the retinal screening done and I’m going to refer her to Desmond as well.”*	2	2
Knowledge	I do not know the guidelines, national or local, for managing type 2 diabetes.	*“I don’t think I’ve ever read the NICE guidelines [for diabetes]”*	14	27
	I know how to manage type 2 diabetes in people with SMI.	*“…when we trained I think our training was very, very helpful in the sense that we did what they called Project 2000, which meant prior to specialising in mental health you had to do all the other areas of nursing. So fortunately I was in A&E, diabetes was one of the things that people come with in A&E so I think my knowledge grew from them.”*	7	13
Memory, Attention & Decision Processes	Managing type 2 diabetes in someone with SMI is a routine part of my job.	*“Completely routine part of the job. As soon as you see somebody who has diabetes you begin that process of finding out what they’re taking, when they’re taking it, how well is it managed and who are they seeing.”*	7	8
	I tailor the treatment of type 2 diabetes in people with SMI depending on their needs.	*“…in the community is that treatment is very often tailored towards the mental health side. So we would be looking at things like risk. We would obviously follow NICE guidelines but we may have to tweak the NICE guidelines for issues like weight, risk of hypoglycaemia and the risk that somebody may be threatening suicide so we don’t want to give them any treatment that’s going to worsen.”*	6	17
Optimism	I am optimistic that I will be able to manage type 2 diabetes in people with SMI.	*“I’ve done it in the past, I’m doing it now, I’m certainly going to do it in the future, yeah.”*	8	10
	I do not feel optimistic about the health of my patients with type 2 diabetes and SMI	*“there’s no point in us thinking we’ve fixed them while they’re in hospital because once they’re feeling well, the same as all of us, once we’re well what do we do? We stop taking our medication.”*	1	1
Reinforcement	I would be disciplined if I did not manage type 2 diabetes in people with SMI	*“I think I’d be disciplined if I’m found to be negligent”*	9	9
	Incentives, such as CQUINS or QOF points, encourage me to manage type 2 diabetes in people with SMI.	*“For organisational reasons we as GPs are incentivised as QOF, quality outcomes from work so referring to diabetic education is a mandatory part of that. So we all do that anyway because we’re incentivised to.”*	8	17
Skills	Managing type 2 diabetes in people with SMI requires special communication and negotiation skills.	*“Yes, I do. I think you need to have a psychological, think, the way, think in a psychological way. So you mustn’t go, you mustn’t be in concrete. Why haven’t you gone to your retinal screening? You need to go, you could go blind is not going to work with someone who’s paranoid and doesn’t want to sit in a dark room with someone staring deeply into their eyes. It’ll just reinforce their fear and they’ll definitely avoid it and they won’t come and see you again. And I’ve seen patients who do that……So I think that everybody who works with this group of people should have some training in, I don't know, maybe not [motivational interviewing] because I think that’s probably too simple but more about trying to explore with them why it is that they haven’t done what it is that they should be doing.”*	12	23
	I need more training in diabetes in order to manage type 2 diabetes in people with SMI	*“Feeling I am purely a mental health nurse by profession so the little bit of general that I did I did a very, very long time ago. To be able to get updates and actually get more training to actually feel confident and there is now the training that was mentioned earlier in the office.”*	9	21
Social influences	I work as part of a team of healthcare professionals to help manage type 2 diabetes in people with SMI	*“…if I’ve got concerns about any cases, part of that clinical meeting is about bringing any concerns, so that could be either about somebody’s mental health or physical health or it could be a social issue that somebody’s experiencing. So I’ve got that avenue as well, so I can get other colleagues’ opinion, well I’ve dealt with that problem before so this is what I would do.”*	15	36
	Family members and carers help me manage type 2 diabetes in someone with SMI.	*“I suppose it depends on the family relationships, not everybody’s got a wonderful supportive family. Not everybody’s got a family that believe that mental illness exists and not everybody’s got a family that believe that diabetes is something that needs treatment. But I think when you’ve got that and you’ve got a patient who does have a level of awareness or a family member, definitely you use that.”*	3	6
	My patients' level of engagement is a key factor in how I manage their type 2 diabetes	*“I guess it’s challenging depending on where they are at that particular moment in time. So on admission if they’re manic you’re not going to engage. If they, by the time they’re leaving the ward and their anti-psychotic medication is working and they’ve got better insight then it’s going to be completely different. Absolutely different attitude altogether.”*	16	69
Social Professional Role & Identity	General practice should take overall responsibility for managing diabetes in people with SMI.	*“Because we’re in the community and the patients are supposed to be, in the community looked after by a GP, so I think the way services are set up is that yes, we can support, but I don’t, I think the identification and the continuous treatment and all that should really stay with the GP”*	15	41
	It is my responsibility to ensure that my service users with diabetes and SMI are able to access the relevant diabetes services.	*“They won’t routinely go for health screenings unless they are really pressed to do so. So, therefore, things can be missed so if we’re the only people they’re seeing, we do have a responsibility to make sure that at least we’re giving them some support to access services.”*	12	22
	I monitor, or help my patients to monitor, blood glucose levels in people with diabetes and SMI.	*“Well, obviously ensuring that they are taking, they’re having, they’re taking their whatever medicines it is for their diabetes, they’re compliant. We are supposed to be doing the, everybody that attends the clinic, doing regular blood sugar monitoring”*	12	24
	I support and advise my patients with diabetes and SMI to lead a healthy lifestyle.	*“But because we see them on a regular basis we can actually support them in attending their appointments and eating healthy and all that, yeah.”*	11	25
	All healthcare professionals are responsible for managing diabetes in people with SMI.	*“It’s everybody from social therapist, care assistants, anybody who has any contact. So their away day doesn’t start with nurses and registered nurses and managers and physical health leads. It starts with the people who are going to be on the ground. It could be the social therapist”*	9	11
	Mental health professionals have a responsibility to understand and monitor diabetes.	*“Well, the care coordinators but yeah, part of their role is to look at the total care of the patient. So they would obviously be expected to be aware that the client has diabetes. I think probably just monitoring that they’re taking medication and that they’re not particularly unwell through the diabetes”*	8	13
	Part of my role is to start new or step up the treatment of diabetes in people with SMI.	*“Well, within the boundaries of what I can do. I wouldn’t, I would never start people on anti-diabetic medication, I would never mess around with a diabetic management, because it’s not what I do on a day to day basis.”*	6	8
	I monitor diabetes medication adherence in people with diabetes and SMI.	*“Well, obviously ensuring that they are taking, they’re having, they’re taking their whatever medicines it is for their diabetes, they’re compliant.”*	6	7
	I monitor, or help my patients to monitor, blood pressure in people with diabetes and SMI.	*“…if I’m in a room that allows it, I will do things like blood pressure, weight, height, calculate BMI. All those sorts of things”*	5	5
	I monitor the weight of people with diabetes and SMI.	*“And I’m, so I’m going back to the depot clinic where we check weight, blood pressure, height, BMI and we are supposed to document new results.”*	4	4
	I assist my patients with diabetes and SMI to attend their diabetes appointments.	*“Well I guess recommending patients attend their annual physical health check to include blood sugar, is something that I do pretty routinely tell my patients so.”*	4	5

### Belief statements

A total of 131 belief statements were created across the 14 domains, ranging between 1 and 20 per domain (mean = 9.4, SD = 6.6). See Additional file 2 for full details of all 131 belief statements and the frequency with which they were coded. After discussion within the research team 42 belief statements, between 1 and 11 per domain, were deemed most relevant. Table 1 details these 42 beliefs, with example anonymised quotations taken directly from the transcripts. The key beliefs within each of the domains of the TDF are summarised below.

### Behavioural regulation

Eight healthcare professionals had developed strategies to help them in managing their behaviour. Five participants had developed and were using action plans, either within their clinical teams or individually, to aid them in managing type 2 diabetes in people with SMI. Another three participants felt that reflecting on and reviewing individual patient care, as part of clinical meetings, was a useful learning exercise enabling healthcare professionals to identify ways to improve their future practice.

### Beliefs about capabilities

Of the 16 participants, nine explicitly stated that they felt confident about their ability to manage diabetes in people with SMI. No participants reported concerns about their capabilities to manage diabetes in this population.

### Beliefs about consequences

Participants expressed a range of beliefs about the potential consequences of not managing diabetes in people with SMI. Many identified that failure to manage the condition effectively would lead to suboptimal diabetes control and as a consequence serious complications including early mortality, amputation, sight loss, diabetic ketoacidosis, kidney failure and stroke. Suboptimal diabetes control was also thought to affect a person’s mental health; this was either because high blood glucose levels led directly to changes in mood and psychotic illness, or because low self-esteem and confidence in managing their condition led to feelings of depression.

### Emotion

Participants expressed concern and worry about managing type 2 diabetes in people with SMI. Concern was expressed in relation to what would happen to service users outside of their contact with a healthcare professional – would they be able to manage their diabetes or would something serious happen to them? Respondents were also worried that even small changes in a service user’s diabetes, or asking clients to make minor changes to their lifestyle could trigger a mental health crisis. This was of particular concern when the individual lacked capacity or understanding, or was experiencing difficult social and personal circumstances, such as living in temporary accommodation or experiencing financial struggles. Frustration was expressed by some healthcare professions in relation to service users and other colleagues not wanting to engage and a lack of appropriate systems to support them in managing diabetes in people with SMI. Fear of mental illness was expressed by only one participant.

### Environmental context and resources

Environmental context and resources were identified as a significant barrier to managing diabetes in people with SMI. Access to an integrated IT system between mental and physical health services was seen as an important way in which more unified care could be delivered, e.g. through improving access to test results. A majority of healthcare professionals also felt that time was a limiting factor. Working with this client group was thought to be particularly challenging and complex; as a result more time was needed to establish a rapport and engage with service users, which might include flexible working and more home visits. Having access and clear care pathways to other services such as primary care, and specialist mental health and diabetes services enabled healthcare professionals to deliver better care, and allowed them to refer service users easily when required.

### Goals

No participant saw the management of diabetes as a greater priority than the management of a service user’s mental health. Healthcare professionals reported that either the management of diabetes and SMI were equally important, or that prioritisation was given to the management of a service user’s mental state. Participants felt that the organisation they worked for had a clear focus and aim to improve management of diabetes in this population. When speaking about the diabetes goals and targets healthcare professionals established with service users, these were often tailored or individualised to the specific needs of someone living with an SMI. Goals needed to be realistic, both from the service user and the healthcare professional perspective, given the population’s potentially limited capacity, understanding and cognitive ability. This often involved focusing on just one target behaviour, or lowering their target HbA1c to a more realistic level.

### Intentions

No participants expressed an explicit intention to manage diabetes in people with SMI. Two participants intended to refer people with diabetes and SMI on to either community exercise programmes, or diabetic retinopathy screening and structured diabetes education.

### Knowledge

There was a clear distinction between participants who were specialist mental health professionals and those who were either primary care or diabetes specialist clinicians in their awareness of guidelines for managing type 2 diabetes. Half of the sample, overwhelmingly mental health professionals, were not aware of or felt they had no understanding of the guidelines for managing diabetes. Six of the seven primary care or diabetes specialist clinicians reported being aware of diabetes guidelines, such as those developed by the National Institute for Health and Social Care Excellence (NICE) in England and Wales, and using them when managing diabetes in people with SMI albeit in an adapted or tailored format. Whilst four healthcare professionals, from a range of professions, reported knowing how to manage diabetes in people with SMI, three mental health professionals felt that their lack of knowledge prevented them from managing diabetes effectively.

### Memory attention and decision processes

Seven participants, across services, described the management and monitoring of type 2 diabetes in people with SMI as a routine part of their role. Many described a degree of automaticity when encountering a service user with diabetes. This included all of the GPs within the sample. Six of the sample made treatment decisions tailored to the individual needs of the service user.

### Optimism

A feeling of hope and optimism about being able to manage type 2 diabetes in people with SMI in the future was expressed by five mental health professionals and one GP. Interestingly one interviewee expressed a lack of hope about the future health of service users with diabetes, particularly in regards to service user’s ability to manage their diabetes once they had been discharged from inpatient care.

### Reinforcement

A majority of healthcare professionals, from a range of professions, expected to be disciplined if they did not effectively manage and monitor diabetes in their service users, either by their employer or professional regulatory body. All three GPs along with two consultant psychiatrists felt that incentives do or would encourage them to manage diabetes in people with SMI, these were primarily pay-for-performance schemes such as the Quality Outcomes Framework (QOF) [33] and Commissioning for Quality and Innovation (CQUIN) [34] targets.

### Skills

Eleven participants felt that being able to manage type 2 diabetes in people with SMI required specialist communication and negotiation skills, in comparison to those without SMI. These skills included enhancing service user’s confidence in their ability to manage their diabetes, knowing how to understand and communicate with people with a range of cognitive abilities, knowing how to develop meaningful relationships with service users and how to negotiate targeted goals. These skills are likely to be required across the diabetes population, but were deemed vital when attempting to engage someone with SMI. As well as specialist skills, nine participants, primarily those from mental health services, felt that they needed more training in how to manage diabetes effectively either before they could undertake this as part of their role or to enhance the care that they delivered.

### Social Influence

All participants, except one psychiatrist, described working collaboratively with healthcare professionals from different disciplines to manage diabetes in people with SMI. The manner in which diabetes was managed was often informed by other members of the team, particularly their mental or physical health counterparts. Mental health staff often sought advice and were informed by GPs and those with specialist diabetes knowledge, and those from primary care and diabetes services were influenced by advice and guidance from their mental health colleagues. Of these 15 participants, three mental health professionals also described family members and carers helping them to manage and monitor diabetes for their loved ones.

The degree to which service users could engage and communicate with their healthcare provider played an important role in whether healthcare professionals attempted to manage diabetes in this population. This manifested in a number of ways; some service users refused to engage with specific healthcare professionals about their diabetes, service users were reported to refuse diabetes treatment altogether or deny their diagnosis, and a lack of insight and in some cases a lack of mental capacity and language barriers often made communication and engagement difficult.

### Social professional role and identity

Participants’ perceived role in the management of type 2 diabetes in people with SMI, and the role of other healthcare professionals and service users, was mentioned by all. Although a majority felt that it was the role of general practice to take overall responsibility for the care of diabetes in this population, half felt that either mental healthcare professionals, including mental health nurses and psychiatrists had a responsibility to understand and monitor diabetes in those within their care or that all healthcare professionals who had contact with service users should take some level of responsibility for managing diabetes.

In reference to their own specific roles, a majority of participants felt that it was their role to ensure the population were able to access relevant diabetes services, support service users to lead healthier lifestyles and either monitor or help service users to monitor their blood glucose levels. It was primarily those from primary care and specialist diabetes services that felt that starting new or titrating diabetes treatments in people with SMI was their responsibility. Whilst mental health professionals felt that monitoring medication adherence, blood pressure, weight and assisting service users to attend their diabetes appointments were their responsibility.

## Discussion

In using the TDF [25], our study has provided a comprehensive understanding of the enablers and barriers to delivering effective care for type 2 diabetes in people with SMI, from the perspective of various healthcare providers. The importance of all theoretical domains illustrates the complexity of diabetes management in this population. Informed by the Medical Research Council (MRC) guidelines [35], use of the TDF provides a foundation for developing a theoretical- and evidence-based intervention by providing a basis on which to model these relationships in a larger sample of participants and subsequently select key behaviour change techniques to be implemented in an intervention.

Knowing how to manage and monitor type 2 diabetes was a significant barrier to implementation. There was poor awareness, particularly amongst mental health professionals, about national and local guidelines for managing type 2 diabetes. As a consequence training on effective diabetes management was identified as a need by all mental health professionals, not just mental health nurses as reported elsewhere in the literature [22]. Theoretical- and evidence based diabetes educational interventions for mental health professionals are scarce. A single group pre-post study [36] reporting the development and evaluation of a diabetes educational package for mental health nurses found significant improvements in diabetes knowledge. However, the quality of the evaluation was poor with non-validated measures, small sample size and no pre-defined hypothesis or power calculation employed.

In order to engage service users in their diabetes care it was recognised that specialist communication and negotiation skills were required, and training needed. The inability of some service users to engage or have insight into their illness and treatment was seen as a significant barrier and therefore development of these skills was deemed vital to engaging the population. Difficulties engaging service users due to limitations in cognitive and executive functioning are also a barrier to preventing diabetes in people with SMI [37, 38]. These findings indicate a clear need for educational and skills based training in order to develop competences in effective communication and behaviour change, as well as implementation of guidelines for managing type 2 diabetes.

As found in the broader diabetes literature [39, 40] time constraints were seen as a significant barrier to delivering effective diabetes care to people with SMI. Despite these time constraints a majority of the sample did however, acknowledge that certain aspects of diabetes care were within their professional role. These specific roles and responsibilities did differ by profession. All participants saw it as their role to refer service users on to relevant diabetes-related services; support and educate people to manage their diabetes and lead healthier lifestyles; and monitor or help service users to monitor their diabetes.

Diabetes medication initiation and titration was deemed solely the responsibility of primary care and specialist diabetes services, as reported by mental health nurses elsewhere in the literature [22]. Whereas, mental health specialists were clear about the importance of physical health monitoring and their role in this, suggesting a clear demarcation of responsibilities, rather than the suggested confusion and role ambiguity [20].

There does however, seem to be discrepancy between participants’ identity as a professional within their workplace, and individual goal priorities. Whilst participants were clear about their role and the policies of the organisation, diabetes was not considered a priority over the management of mental health by any of the sample, with mental health more often the primary focus. The concept of goal priority, the likely prioritization of one goal over another, has been relatively under-researched in relation to health-related behaviours. Recent research does however indicate that when levels of goal priority are high, the intention-behaviour relationship is stronger [41]. This is particularly salient for this study, as participants lacked any intention to manage type 2 diabetes in people with SMI. Although an explicit lack of intention should be addressed by developing or increasing intentions [42], such endeavours are unlikely to lead to changed behaviour unless establishing diabetes management as a healthcare priority is also achieved.

Reviewing past behaviour, either as individuals or part of their clinic teams, helped participants to manage and monitor diabetes. These reviews tended to be in relation to poor performance or practices, in order for staff to reflect, learn and then develop more appropriate action plans for future efforts. Encouraging healthcare professionals to focus on past successes is a mechanism for increasing perceived behavioural control [43], but there has been little investigation of how reflecting on past failures may also be beneficial. This study indicates that doing so may enable healthcare professionals to develop action plans that are more relevant to their individual practices and past performances. Action planning, one of the most prolific behaviour change techniques, has been found to be effective in changing behaviour across a range of contexts [44], and could therefore be a route to improving the care offered to this population.

Integration of mental and physical health care services had the potential to be both a barrier and enabler to delivering diabetes care to people with SMI. This was either as a result of poorly integrated IT systems that prevented easy referrals between services or the inability to access patient healthcare records in order to confirm diagnosis, obtain test results or communicate with other members of the healthcare team. Being able to easily refer service users to other diabetes-related services as a result of good organisational structures was an enabler to effective care. Lack of cooperation between psychiatry and primary care and poor knowledge about the organisation of diabetes care have previously been identified as barriers to preventing diabetes in people with SMI [37, 38]. The current data suggests that when integration and easy access existed this allowed healthcare professionals to seek support and advice from their respective physical and mental health colleagues. The nature of this integration and access was however unclear, and since co-location of mental and physical services seems to have variable impact [19], it maybe not just be proximity that prevents integration but other barriers to engaging healthcare professionals in integrated care, which need further exploration.

Supportive family members and carers made it easier for healthcare professionals to manage type 2 diabetes in people with SMI, as found in the prevention of diabetes [37, 38]. This is in line with recommendations that mental health services employ the Triangle of Care initiative [45], which encourages mental health organisations to ensure staff involve and work closely with family members and cares, where possible, to improve care.

Feelings of hope and optimism about managing diabetes and the health of service users were mixed. This may be an important focus for interventions aimed at health professionals as hope and optimism for the future have been identified as important concepts in the facilitation of recovery in people with SMI [46] and may potentially impact on other health behaviours. There may be a complex interaction between hope and recovery with an important role for social context and interpersonal relationships, particularly for those healthcare professionals who occupy a powerful position in relation to service users' hope. Healthcare professionals must carefully consider how they communicate their own hopefulness about people’s recovery [47], including how they ‘hold’ hope for service users during times of struggle and despondency.

Expecting negative physical and psychological outcomes in patients if diabetes was not effectively managed acted as an enabler to action. Fear of discipline, if anything should occur as a result of absent or poor care, and pay-for-performance schemes were thought to be important reinforcing factors. Blame and punishment are felt by healthcare professionals to be part of health service culture, particularly when someone is involved in an error, near miss or incident [48]. However, considering that the evidence for pay-for-performance systems, such as QOF, in changing healthcare professional behaviour is limited and for their efficiency scarce and inconclusive [49–51] these beliefs appear to be unsupported in the literature.

There are several limitations to our study. Type 2 diabetes is a complex condition that requires a range of monitoring and management; as a result participants were reflecting on a range of targeted behaviours. In order to explore how these beliefs impact on specific diabetes-related behaviours, work is currently underway to understand the enablers and barriers to specific diabetes-related behaviours in a larger sample of healthcare professionals, using these beliefs statements as the basis for a large scale survey. The qualitative nature of the study precludes generalisability to the wider population, as does sampling within one inner city service provider. The snowball recruitment strategy may also mean that healthcare professionals particularly interested and involved in the management of diabetes in people with SMI were approached to take part, which may have biased these findings. Although the sample contained small numbers of each professional group, saturation of themes was achieved. As opposed to the actual causes of behaviour these findings reflect participants’ perceptions of the barriers and enablers to implementing the target behaviours [52], which may not necessarily translate into performance of the behaviour [53]. Finally, there was significant variation in inter-rater reliability. Although use of the TDF provides significant advantages including its theoretical underpinnings, synthesis of concepts from a range of behaviour change theories and systematic approach to identifying a broad range of barriers and enablers [54] it comes with challenges. A lack of clear operational definitions for each of the domains [54] often makes coding of the transcripts difficult, particularly for those without a background in psychology. This is likely to explain the variation in inter-rater reliability found within the study.

## Conclusions

The findings of this study indicate a need for individual and organisational level interventions to support physical and mental health professionals in the delivery of diabetes care for people with SMI. At an individual level, increasing knowledge and optimism, improving skills and supporting healthcare professionals to develop action plans and intentions for their own practice could be effective in addressing disparities in care. At an organisational level, creating links and pathways between mental health and physical services to promote integrated care, prioritizing diabetes within mental health settings and clearly defining the roles and responsibilities are identified as potentially important avenues for intervening. Future work will involve exploring these specific barriers and enablers in a larger sample of healthcare professionals in order to model these outcomes more robustly in line with the MRC guidance [35] for developing complex interventions.

## Abbreviations

CQUIN, commissioning for quality and innovation; GP, general (medical) practitioner; MRC, Medical Research Council; NICE, National Institute of Health and Social Care Excellence; QOF, quality outcomes framework; SMI, severe mental illness; TDF, theoretical domains framework

## Additional files

Additional file 1: Interview Schedule. (DOCX 16 kb) Additional file 2:
**Table S2.** All belief statements by domain. (DOCX 23 kb)
