# Supplementary material for: Implementation of recommended type 2 diabetes care for people with severe mental illness – a qualitative exploration with healthcare professionals
Source: BMC Psychiatry. 2016 Jul 8;16:222. doi: 10.1186/s12888-016-0942-2 (PMC4938935; doi:10.1186/s12888-016-0942-2)
Supplement: Additional file 1: — Interview Schedule. (DOCX 16 kb) [file 12888_2016_942_MOESM1_ESM.docx]

## Interview schedule

Introduction: Thank you for agreeing to take part in this interview. We are conducting this study to help us understand how diabetes is managed in people with severe mental illness. We’re interviewing health professionals across several different specialties so while you may care for many other groups of patients, we’d like to focus on the care of people who have both diabetes and SMI. Some of the questions may appear to be quite similar or obvious to answer but we are trying to understand the topic from a variety of perspectives so bear with me, take your time and answer as frankly as possible. Are you happy to get started?

A. SOCIAL AND PROFESSIONAL ROLE

- I’d like to start by asking how much of your work involves looking after people with diabetes and SMI?
- About how many of your patients do you think would have diabetes and SMI?
- What aspects of diabetes care do you see as part of your role?

Prompts: care planning, education, dietary advice, checking blood glucose levels, BP, weight, cholesterol, kidney function, feet, providing emotional support, ensuring patient has had retinal screening, smoking cessation advice/support

- Which other health professionals do you think should be involved in managing diabetes in people with SMI?

B. KNOWLEGDE

- How would you know whether or not your patient had a diagnosis of diabetes and SMI?
- Tell me about your diabetes management for people with SMI – when a patient with a SMI who also has diabetes comes to you, what do you do?
- Do you think the NICE guidelines for diabetes fit for when managing a person who also has SMI?

C. SKILLS

- Do you think there are any special skills or expertise needed to manage patients with diabetes and severe mental illness?
- What experience do you think is needed to manage patients with diabetes and severe mental illness?
- How easy or difficult do you think it is to manage a patient with diabetes and severe mental illness?

D. BELIEFS ABOUT CAPABILITIES

- How confident are you about managing diabetes in people with severe mental illness?

E. OPTIMISM

- How optimistic are you that in the future you will be able to implement NICE diabetes guidelines for people with SMI?

F. BELIEFS ABOUT CONSEQUENCES

- What do you think might happen to the patient if YOU didn’t take steps to manage the diabetes of someone with SMI?
- What do you think might happen to you if YOU didn’t take steps to manage the diabetes of someone with SMI?
- What do you think might happen to other members of your team if YOU didn’t take steps to manage the diabetes of someone with SMI?

G. REINFORCEMENT

- Is there anything that would encourage or discourage you from managing the diabetes of someone who has a SMI?

Prompts: QoF, CQINs, incentives, sanctions, appraisal, care review

H. INTENTIONS

- Do you think you will effectively manage the diabetes of people with SMI in the future?

I. GOALS

- Considering all the things you have to do when you are managing someone with a severe mental illness, how important is managing their diabetes? Why?

J. MEMORY, ATTENTION AND DECISION PROCESSES

- What thought processes might guide your decision to manage a person’s diabetes?

Prompts: What goes through your mind? Is it a routine part of the job or is it something you need to take time to think about?

K. ENVIRONMENTAL CONTEXT AND RESOURCES

- What factors in your working environment influence whether you are able manage diabetes in someone with severe mental illness?

Prompts: the existence of trained staff, too busy, don’t know how to, other priorities

L. SOCIAL INFLUENCES

- Would other members of your team influence how you manage the diabetes of a person with severe mental illness?

Prompt: what about the [GP, Practice Nurse, DSN, CMHN, Psychiatrist] - do you discuss the management of patients with diabetes and SMI with them? How might those discussions affect your management?

M. EMOTION

- Does managing diabetes in someone with SMI worry or concern you?

N. BEHAVIORAL REGULATION

- If you wanted to change the way you manage diabetes in someone with severe mental illness, how would you do this?

O. PATIENT POPULATION

- How do you engage with patients with diabetes and SMI to help them take an active role in managing their diabetes?

Close: That’s all the questions I have for you, has anything occurred to you about this topic during the interview that we haven’t discussed?

Thank you for taking part.
