# Supplementary material for: Implementation of recommended type 2 diabetes care for people with severe mental illness – a qualitative exploration with healthcare professionals
Source: BMC Psychiatry. 2016 Jul 8;16:222. doi: 10.1186/s12888-016-0942-2 (PMC4938935; doi:10.1186/s12888-016-0942-2)
Supplement: Additional file 2: — Table S2. All belief statements by domain. (DOCX 23 kb) [file 12888_2016_942_MOESM2_ESM.docx]

Additional file 2: Table S2. All belief statements by domain

| **Domain** | **Specific belief** | **No. of participants** | **Total no. of quotes** |
| --- | --- | --- | --- |
| Behavioural Regulation | I have a plan, either on my team or on my own, when managing diabetes in someone with SMI. | 5 | 8 |
|  | I review how I manage diabetes in people with SMI, and identify ways in which I can improve. | 3 | 3 |
| Beliefs about Capabilities | I feel confident about managing diabetes in people with SMI. | 9 | 10 |
| Beliefs about Consequences | If I didn't take steps to manage diabetes in someone with SMI, they would come to serious harm. | 13 | 22 |
|  | Poorly controlled diabetes affects a person’s mental health. | 9 | 9 |
|  | Certain antipsychotic medications make diabetes worse. | 9 | 12 |
|  | With good lifestyle and weight loss you can cure diabetes. | 1 | 2 |
|  | There is no benefit to blood glucose testing in people SMI whose diabetes is stable. | 1 | 1 |
|  | Self-monitoring blood glucose in people with diabetes and SMI can lead to depression. | 1 | 1 |
|  | Providing advice about a healthier lifestyle (i.e. diet and exercise) has the biggest impact on diabetes in people with SMI. | 2 | 3 |
|  | Involving service users in decisions regarding their diabetes treatment encourages better adherence. | 1 | 1 |
|  | If I didn't manage diabetes in people with SMI, they would get lost in the system. | 1 | 1 |
| Emotion | Managing type 2 diabetes in people with SMI worries or concerns me. | 10 | 13 |
|  | I would feel guilty if I didn't manage diabetes in someone with SMI. | 5 | 5 |
|  | Managing type 2 diabetes in people with SMI frustrates me. | 3 | 5 |
|  | Working with people with SMI scares me. | 1 | 1 |
|  | I makes me angry when someone with SMI does not take care of their diabetes. | 1 | 1 |
| Environmental Context & Resources | I have access to a GP to help me manage diabetes in people with SMI. | 13 | 31 |
|  | My trust provides training on how to manage diabetes in people with SMI. | 10 | 13 |
|  | More integrated IT systems would make it easier for me to manage diabetes in people with SMI. | 10 | 18 |
|  | It is easier to manage diabetes in someone with SMI in an inpatient setting. | 10 | 16 |
|  | I don't have enough time to manage diabetes in people with SMI. | 10 | 15 |
|  | The mental health act can be used to ensure a person with SMI receives treatment for their diabetes. | 8 | 11 |
|  | Multidisciplinary care of patients with diabetes and SMI is important for good diabetes management. | 8 | 13 |
|  | I have access to people with specialist diabetes knowledge to help me to manage diabetes in people with SMI. | 11 | 27 |
|  | I have access to someone with specialist mental health knowledge to help me manage people with diabetes and SMI. | 6 | 7 |
|  | I don't have access to the resources I need to manage diabetes in people with SMI. | 6 | 11 |
|  | It is harder to manage diabetes in people with SMI in a community setting. | 5 | 10 |
|  | Staff changes and shortages make it difficult to manage diabetes in people with SMI. | 4 | 6 |
|  | Initiatives are taking place in my trust to improve diabetes care in people with SMI. | 4 | 6 |
|  | I have access to podiatry services to help me manage diabetes in people with SMI. | 4 | 4 |
|  | I have access to a physical health champion or lead to help me manage diabetes in people with SMI. | 3 | 4 |
|  | I have access to a district or primary care nurse to help me to manage diabetes in people with SMI. | 3 | 3 |
|  | I have access to a dietician to help me manage diabetes in someone with SMI. | 3 | 5 |
|  | I have access to retinopathy screening to help me manage patients with diabetes and SMI. | 2 | 2 |
|  | I have access to local guidelines on how to manage diabetes in people with SMI. | 2 | 3 |
|  | I don't have access to physical activity services to help me to manage diabetes in people with SMI. | 2 | 2 |
|  | It is not practical to implement NICE guidelines for diabetes in people with SMI. | 1 | 1 |
|  | I have flexibility about when and where I review diabetes in people with SMI. | 1 | 1 |
|  | I have access to psychological services to help me manage patients with diabetes and SMI. | 1 | 1 |
|  | Healthier food on inpatient wards would make it easier for me to manage diabetes in someone with SMI | 1 | 4 |
| Goals | It is important to manage diabetes in people with SMI. | 10 | 12 |
|  | Diabetes should be part of all assessments and the care plan of people with SMI. | 9 | 12 |
|  | Managing patients' type 2 diabetes is as important as managing their mental health. | 5 | 7 |
|  | Diabetes goals and targets need to be tailored for people with SMI. | 5 | 6 |
|  | There is a definite focus in my trust on managing type 2 diabetes in people with SMI. | 4 | 5 |
|  | It is important to educate and empower service users on how to lead a healthier lifestyle and manage their diabetes. | 4 | 7 |
|  | Sometimes a person's finance and living situation take a priority over managing diabetes in people with SMI | 3 | 3 |
|  | I prioritise management of mental health over physical health in people with type 2 diabetes and SMI. | 3 | 7 |
|  | Guidelines on how to manage type 2 diabetes are crucial. | 2 | 3 |
|  | Performing a risk assessment is an important part of managing diabetes in someone with SMI. | 2 | 2 |
|  | The aim of diabetes treatment is to cause the least amount of harm, rather than achieve perfect diabetes control. | 2 | 2 |
|  | Managing diabetes in people with SMI is a step-by-step process. | 2 | 2 |
|  | Understanding the psychological impact of living with diabetes is important. | 1 | 1 |
| Intentions | If one way of managing diabetes in someone with SMI doesn't work I try another. | 3 | 3 |
|  | I ask every patient with SMI whether they have diabetes. | 2 | 3 |
|  | If someone with SMI has had diabetes for a long-time then I check for complications. | 1 | 1 |
|  | I intend to read the NICE guidelines for diabetes. | 1 | 1 |
|  | I intend to attend training in how to manage diabetes in people with SMI. | 1 | 1 |
|  | I do not intend to change the way I manage diabetes in people with SMI. | 1 | 1 |
|  | I intend to follow NICE diabetes guidelines for patients who have type 2 diabetes and SMI | 2 | 2 |
| Knowledge | I do not know the guidelines, national or local, for managing type 2 diabetes. | 14 | 27 |
|  | You need to know someone has diabetes in order to manage it in people with SMI. | 5 | 6 |
|  | I know how to manage type 2 diabetes in people with SMI. | 7 | 13 |
|  | You need to know someone has a mental health problem in order to manage diabetes in people with SMI. | 2 | 2 |
|  | You need to know about mental health in order to manage diabetes in someone with SMI. | 2 | 2 |
|  | I have basic knowledge about diabetes. | 2 | 3 |
|  | You need to know about the complications of diabetes in order to manage diabetes in people with SMI. | 1 | 1 |
|  | You don't need special knowledge to manage diabetes in someone with SMI. | 1 | 1 |
|  | I'm not aware of structured diabetes education for people with SMI. | 1 | 1 |
| Memory, Attention & Decision Processes | Managing type 2 diabetes in someone with SMI is a routine part of my job. | 7 | 8 |
|  | I tailor the treatment of type 2 diabetes in people with SMI depending on their needs. | 6 | 17 |
|  | When deciding how to manage the diabetes of someone with SMI, I take into account their mental health. | 4 | 8 |
|  | The way I manage diabetes in someone with SMI, depends on how severe their diabetes is. | 4 | 4 |
|  | The management of diabetes in someone with SMI is always in the back of my mind. | 3 | 3 |
|  | I tailor the treatment of diabetes in people with SMI depending on the level of risk. | 1 | 1 |
|  | I need to remember the NICE guidelines for diabetes, when I am caring for someone with SMI. | 1 | 1 |
| Optimism | I am optimistic that I will be able to implement NICE guidelines for diabetes in people with SMI | 7 | 11 |
|  | I am optimistic that I will be able to manage type 2 diabetes in people with SMI. | 8 | 10 |
|  | I do not feel optimistic about the health of my patients with type 2 diabetes and SMI | 1 | 1 |
|  | I am unsure whether I will be able to manage diabetes effectively in people with SMI | 1 | 1 |
|  | I am optimistic about the health of my patients with diabetes and SMI | 1 | 1 |
| Reinforcement | I would be disciplined if I did not manage type 2 diabetes in people with SMI | 9 | 9 |
|  | Incentives, such as CQUINS or QOF points, encourage me to manage type 2 diabetes in people with SMI. | 8 | 17 |
|  | Managing diabetes in people with SMI is rewarding | 2 | 2 |
| Skills | Managing type 2 diabetes in people with SMI requires special communication and negotiation skills.  (I need more training in communication and negotiations skills in order to manage type 2 diabetes in people with SMI) | 12 | 23 |
|  | I need more training in diabetes in order to manage type 2 diabetes in people with SMI | 9 | 21 |
|  | It is difficult to manage complex conditions, such as diabetes, in people with SMI | 5 | 6 |
|  | I have acquired the skills to manage diabetes in people with SMI over time and with experience | 5 | 10 |
|  | It is difficult to know how to help someone with diabetes and SMI. | 2 | 3 |
|  | I feel I have the skills to manage diabetes in people with SMI | 2 | 2 |
|  | It is very difficult to help people with diabetes and SMI to stop smoking. | 1 | 1 |
|  | I need training in mental health in order to manage diabetes in people with SMI | 1 | 2 |
|  | I do not find managing diabetes in people with SMI difficult. | 1 | 1 |
|  | Good decision making skills are important when managing diabetes in people with SMI | 1 | 1 |
|  | Giving lifestyle advice to someone with diabetes and SMI is easy. | 1 | 1 |
|  | Compassion is important when managing diabetes in people with SMI. | 1 | 1 |
| Social influences | I work as part of a team of healthcare professionals to help manage type 2 diabetes in people with SMI | 15 | 36 |
|  | Working collaboratively with service users enables me to manage diabetes in people with SMI effectively | 7 | 11 |
|  | Good communication between services ensures effective management of diabetes in people with SMI | 6 | 7 |
|  | A person's social situation can make it difficult to manage diabetes in someone with SMI. | 4 | 4 |
|  | Other members of my teams do not influence how I manage diabetes in people with SMI. | 3 | 3 |
|  | Family members and carers help me manage type 2 diabetes in someone with SMI. | 3 | 6 |
|  | A person's behaviour can affect how I manage their diabetes. | 5 | 5 |
|  | It is harder to manage diabetes in someone with SMI compared to someone without SMI | 3 | 5 |
|  | A person's ethnicity and gender will affect the way I manage diabetes in people with SMI | 2 | 2 |
|  | When I am worried about the diabetes health of a patient with SMI, I have people I can turn to for advice. | 1 | 1 |
|  | Other healthcare professionals make it harder for me to manage diabetes in people with SMI. | 1 | 3 |
|  | Newly diagnosed patients with diabetes are the hardest to manage. | 1 | 1 |
|  | My patients' level of engagement is a key factor in how I manage their type 2 diabetes | 16 | 69 |
| Social Professional Role & Identity | General practice should take overall responsibility for managing diabetes in people with SMI. | 15 | 41 |
|  | It is my responsibility to manage diabetes in people with SMI. | 12 | 34 |
|  | It is my responsibility to ensure that my service users with diabetes and SMI are able to access the relevant diabetes services. | 12 | 22 |
|  | I monitor, or help my patients to monitor, blood glucose levels in people with diabetes and SMI. | 12 | 24 |
|  | I support and advise my patients with diabetes and SMI to lead a healthy lifestyle. | 11 | 25 |
|  | All healthcare professionals are responsible for managing diabetes in people with SMI. | 9 | 11 |
|  | Mental health professionals have a responsibility to understand and monitor diabetes. | 8 | 13 |
|  | I take a holistic approach to managing d5iabetes in people with SMI. | 8 | 19 |
|  | Part of my role is to start new or step up the treatment of diabetes in people with SMI. | 6 | 8 |
|  | I monitor diabetes medication adherence in people with diabetes and SMI. | 6 | 7 |
|  | I monitor, or help my patients to monitor, blood pressure in people with diabetes and SMI. | 5 | 5 |
|  | Part of my role is to educate patients with SMI about diabetes. | 4 | 4 |
|  | I monitor the weight of people with diabetes and SMI. | 4 | 4 |
|  | I assist my patients with diabetes and SMI to attend their diabetes appointments. | 4 | 5 |
|  | Diabetes specialists should take overall responsibility for managing diabetes in people with SMI. | 4 | 7 |
|  | If a patient with diabetes and SMI has a serious problem I refer them to A&E. | 3 | 4 |
|  | I do not perform foot checks on people with SMI and diabetes. | 3 | 3 |
|  | Service users need to take more responsibility for managing their diabetes. | 1 | 1 |
|  | Physical healthcare professionals have a responsibility to understand mental health. | 1 | 1 |
|  | I do not monitor kidney function in people with diabetes and SMI. | 1 | 1 |
